# Trophic complexity alters the diversity–multifunctionality relationship in experimental grassland mesocosms

**DOI:** 10.1002/ece3.7498

**Published:** 2021-03-31

**Authors:** Krishna Anujan, Sebastian A. Heilpern, Case M. Prager, Brian C. Weeks, Shahid Naeem

**Affiliations:** ^1^ Department of Ecology, Evolution and Environmental Biology Columbia University New York NY USA; ^2^ Department of Natural Resources Cornell University Ithaca NY USA; ^3^ Rubenstein School of Environment and Natural Resources University of Vermont Burlington Vermont USA; ^4^ School for Environment and Sustainability University of Michigan Ann Arbor MI USA

**Keywords:** biodiversity loss, ecosystem function, jack‐of‐all‐trades effect, trophic simplification

## Abstract

Plant diversity has a positive influence on the number of ecosystem functions maintained simultaneously by a community, or multifunctionality. While the presence of multiple trophic levels beyond plants, or trophic complexity, affects individual functions, the effect of trophic complexity on the diversity–multifunctionality relationship is less well known. To address this issue, we tested whether the independent or simultaneous manipulation of both plant diversity and trophic complexity impacted multifunctionality using a mesocosm experiment from Cedar Creek, Minnesota, USA. Our analyses revealed that neither plant diversity nor trophic complexity had significant effects on single functions, but trophic complexity altered the diversity–multifunctionality relationship in two key ways: It lowered the maximum strength of the diversity–multifunctionality effect, and it shifted the relationship between increasing diversity and multifunctionality from positive to negative at lower function thresholds. Our findings highlight the importance to account for interactions with higher trophic levels, as they can alter the biodiversity effect on multifunctionality.

## Graphical abstract

We used a manipulated grassland mesocosm experiment to test the effects of higher trophic levels on ecosystem multifunctionality. We find that the number and identity of trophic levels affect the jack‐of‐all‐trades relationship between biodiversity and ecosystem multifunctionality. Our findings have implications in refining predictions for ecosystem multifunctionality in the face of ongoing biodiversity loss.

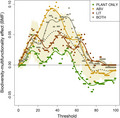

## 1 INTRODUCTION

Biodiversity has a positive effect on the magnitude of individual ecosystem functions and on the number of functions an ecosystem maintains simultaneously (multifunctionality) (Duffy et al. 2017; Hector & Bagchi, 2007; Hooper et al. 2005; Liang et al. 2016; Zavaleta et al. 2010). Empirical support for this diversity–multifunctionality relationship, across taxa and habitats, suggests that higher levels of biodiversity may be necessary to maintain ecosystem functioning than previously assumed based on single‐function studies (Cardinale et al. 2012; Hooper et al. 2005; Lefcheck et al. 2015). Moreover, an increase in total biodiversity in an ecosystem often corresponds with an increase in trophic complexity, which can then alter ecosystem functioning (Haddad et al. 2009; Soliveres et al. 2016). However, the role of trophic complexity in influencing how biodiversity mediates multifunctionality is less well understood.

Nonproducer trophic levels (e.g., litter decomposers, herbivorous insects) can have positive or negative impacts on various ecosystem functions simultaneously (Dyer & Letourneau, 2003; Estes et al. 2011; Naeem et al. 1994, 1995; Schmitz, 2006; Strickland et al. 2013; Tiffin & Ross‐Ibarra, 2014), leading to complex effects on multifunctionality. Depending on the functional role of the group and the ecosystem function considered, nonproducer trophic levels can either directly enhance or reduce the magnitude of function. For example, aboveground herbivores can decrease aboveground plant biomass, while aboveground predators can increase it; litter decomposers can increase decomposition rates and root biomass, while bacterivores can reduce decomposition (Seabloom et al. 2017; Soliveres et al. 2016). Further, nonproducer trophic levels could shift plant resource allocation and functional traits and indirectly affect ecosystem functions by altering biodiversity–ecosystem functioning (BEF) relationships (Burghardt, 2016; Cadotte, 2017; Salgado‐Luarte & Gianoli, 2012). While nonproducer trophic levels can affect several individual functions simultaneously, how these interactions scale up to influence the biodiversity–multifunctionality relationship is poorly understood.

Beyond the cumulative effects of nonproducer trophic levels on single ecosystems functions, overall multifunctionality depends on existing correlations between species contributions to these functions. When ecosystem functions are positively correlated, fewer species are necessary to maintain multifunctionality than when ecosystem functions are negatively correlated with each other (Heilpern et al. 2020). Further, negative correlations among ecosystem functions make it unusual for species to simultaneously maximize the provisioning of multiple functions at high levels. These negative correlations thus lead to the observed pattern where diversity has a positive impact on multifunctionality when lower levels of ecosystem functions are considered and a negative impact at high levels, called the “jack‐of‐all‐trades” effect (van der Plas et al. 2016). However, trophic complexity could affect the magnitude of, and correlation among, species contributions to individual ecosystem functions, causing deviations from the jack‐of‐all‐trades pattern. Disentangling the effects of trophic complexity and plant diversity on multifunctionality has important consequences toward predicting the effects of ongoing, nonrandom biodiversity loss on ecosystem functioning, and mechanistic approaches, such as experiments, are important first steps to make these predictions.

Here, we explore the effects of diversity and trophic complexity on ecosystem multifunctionality using data from an experimental manipulation of plant diversity and trophic complexity on multiple ecosystem functions in tall‐grass prairie mesocosms. This work was conducted at Cedar Creek Ecosystem Science Reserve, Minnesota, USA, between 2000 and 2001, for the purposes of exploring the interaction between diversity and trophic structure on soil fertility. Its design, however, provides a unique opportunity to test the impacts of trophic complexity on the diversity–multifunctionality relationship. The study is a fully factorial design with 5 plant diversity treatments (1, 2, 4, 8, and 16 spp.) crossed with 4 trophic complexity treatments that represent four basic levels: a close to natural community (plants + litter fauna + aboveground mesofauna), two communities with half the functional groups absent (plants + litter fauna and plants + aboveground mesofauna), and a null community with plants alone. We note that species diversity within each level of trophic complexity could potentially affect ecosystem functioning and multifunctionality, but our experimental design cannot disentangle these effects. Four ecosystem functions were measured: aboveground biomass, belowground root biomass, soil water retention, and biomass recovery after harvest in the following year.

**FIGURE 1:**
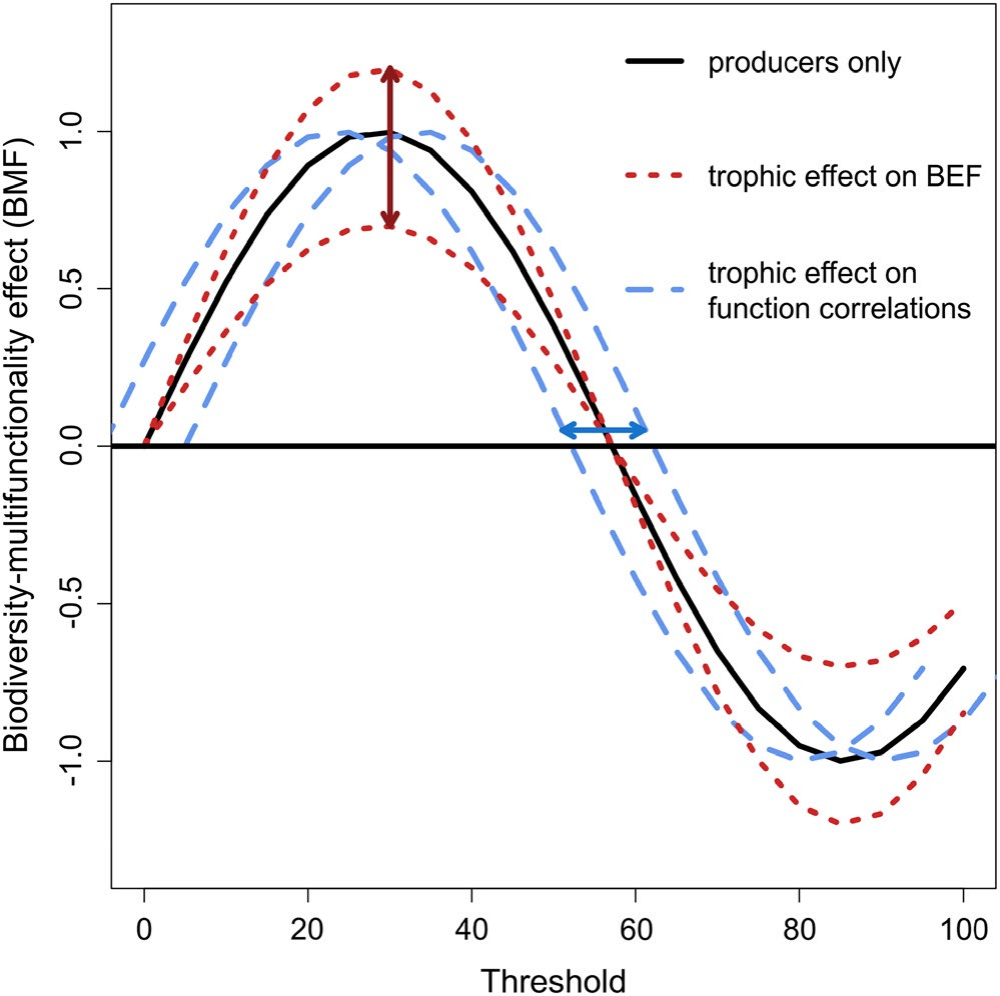
Conceptual framework illustrating hypothesized effects of trophic complexity on the biodiversity–multifunctionality effect (BMF) curve. The BMF is a measure of the slope of the relationship between diversity and multifunctionality (e.g., number of functions gained through the addition of species) whose value is dependent on the threshold (i.e., percent ecosystem function obtained) used in estimating multifunctionality. Positive effects of diversity correspond to positive BMF values, or a curve above zero and vice versa. The continuous black curve represents a hypothetical relationship between selected threshold value and the BMF for a plant community in the absence of trophic complexity (i.e., the plant only curve). The red and blue lines represent possible deviations from the plant‐only curve with the addition of trophic complexity. The red curves represent trophic‐induced changes to diversity effects on single ecosystem functions or BEF, which alter the flatness of the BMF curve. The blue curves represent trophic‐induced changes to correlations between traits, which shifts the horizontal location of the BMF switch from positive to negative

We hypothesized that trophic complexity may impact multifunctionality and the shape of the jack‐of‐all‐trades curve in two ways. First, trophic complexity will affect the slope of the relationship between plant diversity and multifunctionality (i.e., the biodiversity–multifunctionality (BMF) effect) at different levels of ecosystem functioning, consequently resulting in vertical shifts in the jack‐of‐all‐trades curve (Figure 1). This would occur because trophic complexity could alter the magnitude of individual ecosystem functions either directly, through independent effects on functioning, or indirectly, through interspecific interactions that affect BEF curves. Second, trophic complexity will horizontally shift in the jack‐of‐all‐trades curve, especially at the inflection point where it crosses the x‐axis (Figure 1). This pattern could occur through the impact trophic complexity has on correlations between individual ecosystem functions in a community and the values of functions at which diversity has a positive effect on multifunctionality. These potential impacts of trophic complexity therefore result in distinguishable, independent and nonmutually exclusive, shifts in the jack‐of‐all‐trades curve (Heilpern et al. 2020). To test these hypotheses, we model the effects of trophic complexity on (i) individual functions, (ii) multifunctionality at multiple thresholds, and (iii) the biodiversity–multifunctionality (BMF) effect (the gain in number of ecosystem functions maintained above the given value with one additional species) across the range of possible thresholds.

## 2 METHODS

### 2.1 Experimental methods

Tall‐grass prairie mesocosms were established adjacent to the biodiversity experimental sites in the Cedar Creek Ecosystem Science Reserve, Minnesota, USA. The experimental design was factorial with 100 one‐meter‐diameter pots that were inside netted insect exclosures. Each pot was maintained at one of 5 levels of plant diversity: 1, 2, 4, 8, or 16 species. The plant species used in this experiment (Table S1) were native perennial species used in previous experimental studies from the site (Seabloom et al. 2017; Tilman et al. ,^2001^, ^2006^). Pots with incomplete data on species identity were excluded from our analyses, resulting in a sample size of 94. The plant diversity treatments were crossed with trophic complexity treatments, such that pots included plants and aboveground mesofauna, plants and litter mesofauna, plants and both aboveground and litter mesofauna, or plants only.

Following previous studies, these treatments were achieved by first applying a pesticide treatment on all the pots, which initially contained only sterilized local soil and litter, removing all fauna (Seabloom et al. 2017; Tilman et al. 2012). The pesticide used was esfenvalerate (DuPont™ Asana® XL), a natural pyrethrin insecticide, known to have no nontarget effects such as phytotoxicity or fertilization (DuPont, 2002; Mitchell, 2003). The choice of the pesticide was determined by the Cedar Creek Ecosystem Reserve treatment protocols and was similar to other studies such as Tilman et al. (2012); Seabloom et al. (2017). Each replicate was then treated with an extract prepared from a single soil slurry of fresh soil cores from the original site to introduce a standardized community of microorganisms.

Treatments with aboveground fauna were then inoculated with identical sets of invertebrates that included all species obtained from sweep‐netting adjacent vegetation. To control for biomass effects, equivalent sets of frozen, killed invertebrates were added to the treatments without aboveground fauna. Removal of aboveground fauna in appropriate treatments was ensured by monthly pesticide treatments, and a pesticide control was applied to the other plots by spraying equal volumes of water. Similarly, treatments with litter fauna were inoculated with active leaf litter each month (mesh bags filled with 40 g of leaf litter collected from the surrounding field placed on the ground near the mesocosms for a minimum of two weeks). Controls that included autoclaved leaf litter and litter fauna were simultaneously added to treatments with no litter fauna. Appendix Figure S1 summarizes the counts and community compositions of these treatments.

The experiment was established in 1999 and seeded in spring 2000, and then run for a year. In July 2001, the experiment was ended, plants harvested, and aboveground biomass, root biomass, and soil water retention were measured. Further, in 2002, following natural recruitment, the biomass in each pot was measured to assess recovery.

### 2.2 Ecosystem function measurements

Four ecosystem functions were analyzed. In 2001—after one year of the experiment—in each pot, we measured (i) total aboveground biomass, (ii) root biomass (i.e., the total belowground biomass), and (iii) water retention (quantified as the time taken for a fixed volume of water to flow into a collection flask at the bottom of the pot). In 2002, following the harvesting of both aboveground and root biomass, we characterized a fourth ecosystem function: biomass recovery, which was characterized as the total recovered biomass in a pot after disturbance (the removal, in 2001, of the aboveground and root biomass). These four functions were chosen as they represent key properties of ecosystem function and have potential links to trophic complexity. Moreover, we also chose them for low correlations and thus independent contributions to multifunctionality. Pairwise correlations between these show overall low correlations between the functions at the plot level (Appendix Figure S3), with aboveground biomass and water retention most highly correlated (r = 0.32).

### 2.3 BEF curves

All analyses were performed using the statistical software R, version 3.4.4 (Team, 2009). The response of each ecosystem function to manipulated plant diversity and trophic complexity treatment was analyzed as a log‐linear model using generalized linear mixed‐effects models in the package lme4 (Bates et al. 2015). The likelihood of the full model:

$$F_{i}\;\text{log}\left(\text{Plant richness}\right)+\text{Trophic complexity}+\text{log}\left(\text{Plant richness}\right)*\text{Trsophic complexity}+\varepsilon ,$$

was compared using stepwise selection against the likelihoods of reduced models that did not include the interaction term of plant species richness with trophic complexity, and a simple model that did not include trophic complexity, using AIC values.

### 2.4 Biodiversity–multifunctionality effects

To assess multifunctionality across biodiversity treatments, measurements of the four ecosystem functions—aboveground biomass, root biomass, water retention, and biomass recovery after harvest—were analyzed using a threshold approach [for details, see Byrnes et al. 2014; Manning et al. 2018]. To this end, the maximum value for each ecosystem function was calculated as the mean of the five highest function values across all pots. Each ecosystem function in a pot was then standardized between this maximum value and the minimum value found in any pot in the experiment. The function value was set to 1 if higher than the threshold (25, 50, or 75% of the max) and 0 otherwise. Thus, multifunctionality of any given plot represented the number of functions above the threshold. The linear model fits of multifunctionality as a function of plant diversity and trophic complexity were analyzed at 20%, 40%, 60%, and 80%, as per published methodology (Byrnes et al. 2014; Manning et al. 2018).

To analyze the sensitivity of BMF to trophic complexity and threshold, we fitted similar linear models of multifunctionality as a function of plant diversity and trophic complexity for 100 threshold values between the standardized minimum and maximum (0% and 100%). For the model at each threshold value, the slope of a linear model of multifunctionality as a function of the manipulated plant diversity in the community (equivalent to the increase in multifunctionality with one additional species in the community) was defined as the BMF. The change in BMF values across thresholds, the jack‐of‐all‐trades curve, was then analyzed across trophic complexity treatments to examine the magnitude of the peak, and the point at which the curve crosses the x‐axis (Figure 1). The deviations of the jack‐of‐all‐trades curves for the treatments with more than one trophic level from the curves with plants only were tested using pairwise Wilcoxon signed‐rank tests, a nonparametric test for curve comparisons.

## 3 RESULTS

### 3.1 Single ecosystem functions (BEF)

**FIGURE 2:**
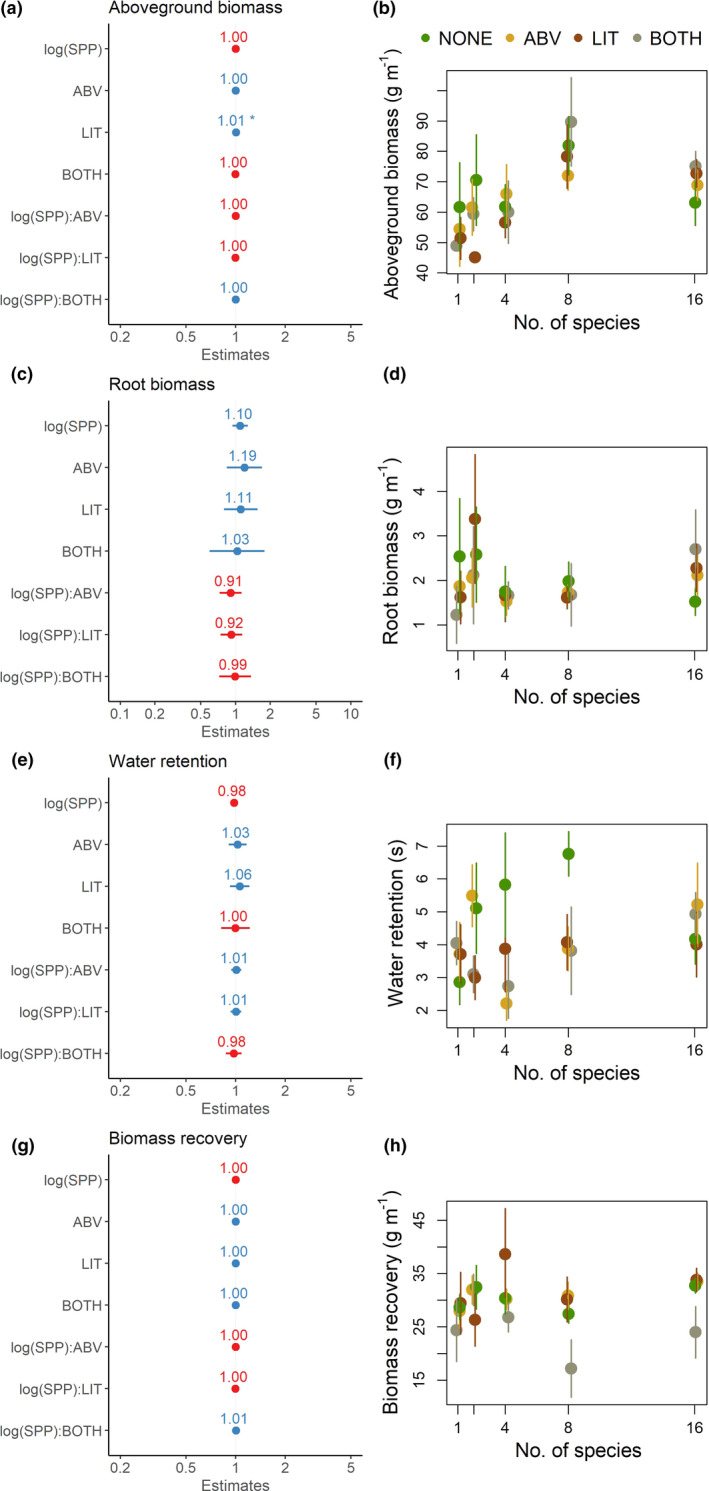
GLM fits of log‐linear models of the four functions—a) aboveground biomass, c) root biomass, e) water retention, and g) biomass recovery as predicted by plant species richness. Log(SPP) is the log of manipulated plant species richness in the experiment. For b), d), f), and h), points show group means and vertical lines show standard errors, and the colors represent the different trophic complexity treatments; only plants (NONE, green), plants and aboveground mesofauna (ABV, yellow), plants and litter mesofauna (LIT, brown), plants and both aboveground and litter mesofauna (BOTH, gray). The points in b), d), f), and h) are jittered along the x‐axis for readability. However, experimental treatments along the x‐axis are 1, 2, 4, 8, or 16 species

In generalized linear models of ecosystems functions, plant species diversity did not have a significant impact on any ecosystem functions measured (Figure 2). In simple models of ecosystem function against plant diversity alone (ecosystem function_1_ ~ log(plant richness)), plant diversity did not have a significant effect on root biomass (R^2^ = 0.0, *p* =.9), water retention (R^2^ = 0.02, *p* =.13), or biomass recovery (R^2^ = 0.0, *p* =.75), but we observed a significant positive saturating effect on aboveground biomass (log(plant richness) coefficient=−0.0017, R^2^ = 0.12, *p* <.001). The total effects of species richness were low, and the average values of each function remained within a small range of values across different plant diversity treatments (Appendix Figure S2).

In the full model with plant diversity, trophic complexity and their interaction (ecosystem function_1_ ~ log(plant richness)+trophic complexity + log(plant richness):trophic complexity), trophic complexity effects on single ecosystem functions were largely nonsignificant, except the litter mesofauna treatment for aboveground biomass (Figure 2). Further, when the effect of trophic complexity was removed using stepwise selection, the best‐fit models for each ecosystem function did not include trophic complexity as a predictor; the simplest model with only plant diversity as the predictor had the lowest AIC.

### 3.2 Biodiversity–multifunctionality (BMF) effects

**FIGURE 3:**
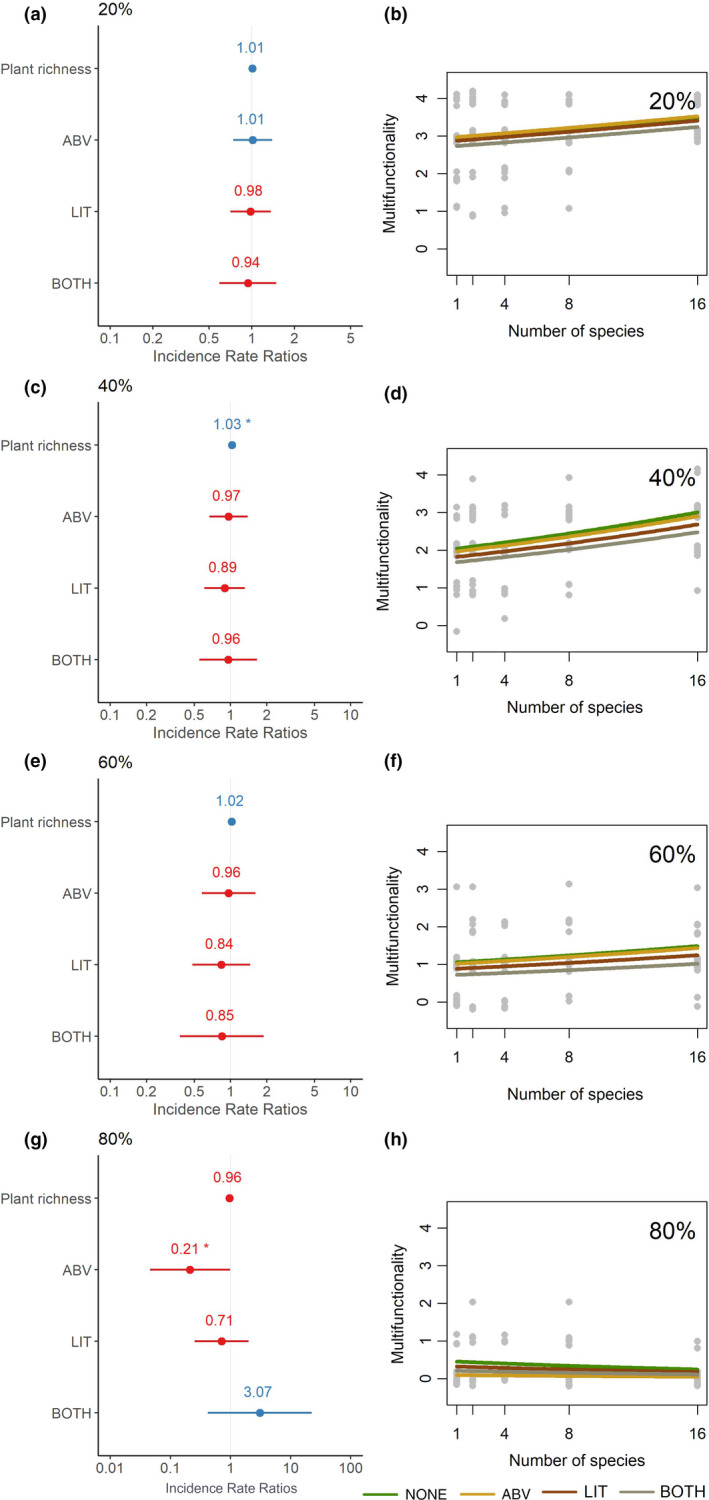
Number of functions above four different thresholds, indicated in the top right corner of each panel, against the number of species in the plot. Lines represent linear model fits for pooled data (black) and each treatment (colors). Legend shows color codes for treatments; only plants (NONE, green), plants and aboveground mesofauna (ABV, yellow), plants and litter mesofauna (LIT, brown), and plants and both aboveground and litter mesofauna (BOTH, gray). Actual data points for each plot represented as gray dots

We found that when multifunctionality was modeled as a function of plant richness and trophic complexity, plant biodiversity was significantly associated with ecosystem multifunctionality at moderate thresholds (at 40%, slope = 0.02, *p* <.05). However, although not significant, the general relationship between plant diversity and ecosystem multifunctionality was positive at low thresholds and negative at high thresholds (Figure 3). Overall, linear models of multifunctionality at a given threshold as predicted by plant diversity and trophic complexity showed that plant diversity had a positive effect at 20% (slope = 0.01, SE = 0.01, *p* =.280), 40% (slope = 0.02, SE = 0.01, *p* =.3*), and 60% (slope = 0.02, SE = 0.01, *p* =.19) but negative at 80% (slope = −0.04, SE = 0.04 *p* =.36) (Figure 3). Moreover, the effect of trophic complexity was significant only at h thresholds; trophic complexity was not a significant predictor at 20% threshold (−0.03, SE = 0.08) and 40% threshold (−0.07, SE = 0.1), but was significant at 60% (−0.20, SE = 0.14) and at 80% (−0.65, SE = 0.31). At higher thresholds, most treatments did not achieve the set threshold function, making a biodiversity or trophic effect difficult to detect.

### 3.3 The jack‐of‐all‐trades curve

**FIGURE 4:**
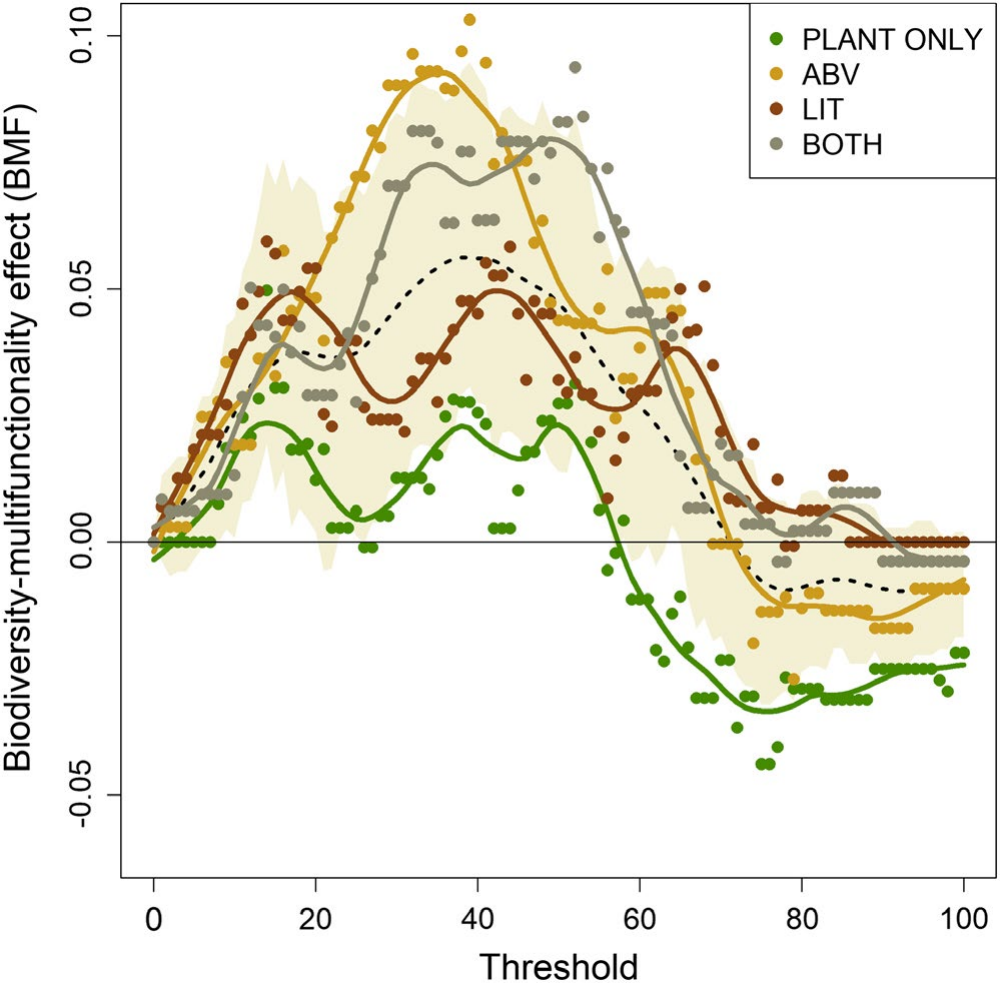
Effect of ecosystem–function threshold on the biodiversity–multifunctionality effect (the BMF). Each point represents the slope (i.e., strength) of the relationship between plant species richness and number of functions above the threshold when estimated using a linear regression model. Each BMF curve for each level of trophic complexity is plotted using a different color, as presented in the key (top right); only plants (PLANT ONLY, green), plants and aboveground mesofauna (ABV, yellow), plants and litter mesofauna (LIT, brown), and plants and both aboveground and litter mesofauna (BOTH, gray). The curves are smooth‐spline interpolations. The dashed line represents the mean slope, and the beige polygon represents the bounds of the standard error of the slope in the pooled dataset

The BMF increased and peaked at moderate thresholds, switching to a negative at high thresholds for all 4 treatments, following predictions of the jack‐of‐all‐trades effect (Figure 4). The relationship of the biodiversity–multifunctionality (BMF) effect to measured threshold was sensitive to trophic complexity (Figure 4). When compared, using the Wilcoxon signed‐rank tests, the BMFs of all multitrophic treatments were significantly different from the plant‐only curve (plants + aboveground mesofauna: W = 8,390, effect size = 0.029, *p* <.01; plants + litter mesofauna: W = 7,808, effect size = 0.03, *p* <.01; plants + aboveground +litter mesofauna: W = 8,149, effect size = 0.03, *p* <.01).

Trophic complexity had an effect on both the height and location of the peak BMF (Figure 4). Across all thresholds, we found that treatments with at least one additional trophic level had consistently higher BMF values than the plant‐only treatment. Among the three complexity levels, treatments with aboveground mesofauna had a higher peak BMF than the treatment with litter fauna as the only additional level. Moreover, the plant‐only and plants + litter mesofauna treatments had no clear peak, while the other two treatments peaked at similar intermediate thresholds (~ 35%– 50%). We also found that treatments with one additional level of trophic complexity transitioned from positive to negative BMFs at higher thresholds ~ 70% – 90% thresholds, than the plant‐only treatment (shift ~ 50% – 60%). Although at high thresholds, differences among treatments with an additional trophic component were less detectable, each remained distinct from the treatment with plants alone.

## 4 DISCUSSION

We find that trophic complexity affects the relationship between biodiversity and ecosystem multifunctionality in two ways. First, the strength of the BMF effect is different across the spectrum of levels of ecosystem function, depending on trophic complexity; treatments with additional trophic levels beyond plants had higher strengths of BMF across thresholds (Figures 3, 4). Second, the shapes of the jack‐of‐all‐trades curves were strikingly different, staying positive for higher thresholds in treatments with more than one trophic level (Figure 4). Together, these findings are indicative of pervasive impacts of trophic complexity on the relationship between biodiversity and ecosystem multifunctionality.

Trophic complexity may alter BMF effects by changing the magnitude of biodiversity effects on individual ecosystem functions; this relationship has been observed in grassland communities similar to ours (Lefcheck et al. 2015; Soliveres et al. 2016). Interestingly, our analyses did not reveal significant impacts of trophic complexity on single ecosystem functions as modeled by BEF relationships (Figure 2, Appendix Figure S2), in contrast with studies on similar landscapes (Soliveres et al. 2016). However, functional groups within mesofauna that were not distinguished in this study (e.g., aboveground herbivores and predators grouped as aboveground mesofauna) could have opposing impacts on individual ecosystem functions. This could make it difficult to disentangle the positive and negative effects of these different groups on multifunctionality. Moreover, the effects of nonproducer trophic levels on plant communities and ecosystem functioning could be latent, delayed, or accruing over time and hence difficult to detect in short‐term manipulations (Maguire et al. 2015; Root, 1996).

Although we did not observe significant impacts of trophic complexity on single ecosystem functions (Figure 2), our results show that the presence of nonproducer trophic levels increases the slope of the biodiversity–multifunctionality curve when examined using a jack‐of‐all‐trades approach (Figure 4). At low thresholds of ecosystem function, treatments that had any amount of trophic complexity amplified the positive diversity–multifunctionality relationship and increased the height of the peak, with the highest slopes at moderate thresholds. This finding is consistent with the observation that increases in plant biodiversity (i.e., single trophic‐level analyses) tend to have the largest impact on ecosystem multifunctionality when moderate levels of ecosystem function are considered (van der Plas et al. 2016). Although we do not see significant trophic complexity effects at specific thresholds when treatments were grouped together(Figure 3), our results with the jack‐of‐all‐trades approach allows a pairwise comparison of the effects of trophic complexity against treatments with plants alone, possibly leading to the observed significant effects. In addition to BEF mechanisms of complementarity and selection, plant diversity is observed to decrease herbivory damage in natural systems (Baraza et al. 2006; Hambäck et al. 2014). This indirect effect of herbivory on plant biomass could also potentially explain the amplified effect of plant diversity on multifunctionality that we observe in the presence of these trophic groups. Thus, it is possible that changes in BEF driven by trophic complexity have resulted in the observed shifts in the BMF curve (Figure 4), although we find no evidence to support this.

Rather, given the impacts of trophic complexity on multifunctionality but the absence of detectable effects on single functions, our results suggest that trophic interactions could mediate BMF by altering trait correlations among plant species, a mechanism observed through numerical simulations (Heilpern et al. 2020). Although independent frameworks to assess identity effects of individual species and environmentally linked intraspecific trait variation in species for BEF and multifunctionality have been proposed (Laughlin, 2014; Meyer et al. 2018), the role of induced trait variation and shifts in function correlations through biotic mechanisms is less explored in the context of multifunctionality. Our experiment was not designed to test trait mechanisms determining BMF, but further explorations would benefit from explicit measurements of plant functional traits across treatments, both in response to trophic complexity and as effectors of ecosystem functioning.

In addition to changes in the amplitude, we also find that trophic complexity shifts the ranges in which the impact of biodiversity on multifunctionality is positive. While biodiversity has been shown to have a positive effect on multifunctionality at low‐to‐moderate values of ecosystem functions and a negative effect at higher values (the jack‐of‐all‐trades effect) in a range of ecosystems, we find that the substantial variation in the inflection point could be driven by trophic complexity (Haddad et al. 2009; Connor et al. 2017; Scherber et al. 2010; Seabloom et al. 2017) (Figure 4). Specifically, the addition of at least one trophic component showed a distinct shift in the threshold at which BMF shifts from positive to negative, with complexity leading to positive BMFs for a higher range of thresholds (Figure 4). Despite the limitations of small sample size, through this study, we observe that both number and identity of trophic groups matter to multifunctionality. A critical direction for future mechanistic studies is to detail how different trophic guilds affect overall multifunctionality.

Our findings have important implications for understanding the relationship between biodiversity and ecosystem multifunctionality. Plant diversity is currently understood to be critical to sustaining multifunctionality at, or below, moderate function threshold values, but our results show that such effects are influenced by trophic complexity. This is particularly true in our most complex—and thus, most realistic—treatment. Global biodiversity loss is occurring across all trophic groups and steep declines in insect populations are widespread (Hallmann et al. 2017). Linking trophic complexity to ecosystem multifunctionality is crucial for improving our predictions of changes in future ecosystem functioning in the face of biodiversity loss. Our results suggest that sustaining a broad spectrum of ecosystem functions and the services they provide will require either sustaining trophic complexity or sustaining greater levels of plant diversity in the face of widespread trends in trophic simplification.

## CONFLICT OF INTEREST

None declared.

## AUTHOR CONTRIBUTION

**Krishna Anujan:** Conceptualization (equal); Data curation (equal); Formal analysis (lead); Methodology (equal); Visualization (lead); Writing‐original draft (lead). **Sebastian Arnold Heilpern:** Conceptualization (equal); Formal analysis (supporting); Methodology (supporting); Visualization (supporting); Writing‐original draft (supporting); Writing‐review & editing (equal). **Case M Prager:** Conceptualization (equal); Data curation (equal); Visualization (supporting); Writing‐review & editing (supporting). **Brian C Weeks:** Conceptualization (equal); Methodology (supporting); Visualization (supporting); Writing‐review & editing (equal). **Shahid Naeem:** Conceptualization (equal); Funding acquisition (lead); Investigation (lead); Methodology (equal); Project administration (lead); Resources (lead); Supervision (lead); Writing‐original draft (supporting); Writing‐review & editing (equal).

## AUTHORS’ CONTRIBUTIONS

SN: Funding acquisition; experimental design and data collection. KA, CMP: Data curation. All authors: Conceptualization of the analyses; contribution to revisions. KA: Formal analysis; initial draft of the manuscript with support from SAH.

## Supporting information

Supplementary Material

## Data Availability

The data from the experiment and the code used for analyses are available through Dryad (https://doi.org/10.5061/dryad.1c59zw3v4).
