# Supplementary material for: Trophic complexity alters the diversity–multifunctionality relationship in experimental grassland mesocosms
Source: Ecol Evol. 2021 Mar 31;11(11):6471–9. doi: 10.1002/ece3.7498 (PMC8207441; doi:10.1002/ece3.7498)
Supplement: Supplementary file 1 — Supplementary Material [file ECE3-11-6471-s001.docx]

Appendix Table 1: List of plant species for the experiment

| Sl No | Species |
| --- | --- |
| 1 | *Achillea millefolium* |
| 2 | *Andropogon gerardii* |
| 3 | *Anemone cylindrica* |
| 4 | *Asclepias tuberosa* |
| 5 | *Buchloe dactyloides* |
| 6 | *Aster azureus* |
| 7 | *Coreopsis palmata* |
| 8 | *Elymus canadensis* |
| 9 | *Euphorbia corollata* |
| 10 | *Koeleria cristata* |
| 11 | *Liatris aspera* |
| 12 | *Panicum virgatum* |
| 13 | *Rudbeckia hirta* |
| 14 | *Schizachyrium scoparium* |
| 15 | *Solidago nemoralis* |
| 16 | *Sorghastrum nutans* |
| 17 | *Sporobolus cryptandrus* |
| 18 | *Bouteloua gracilis* |


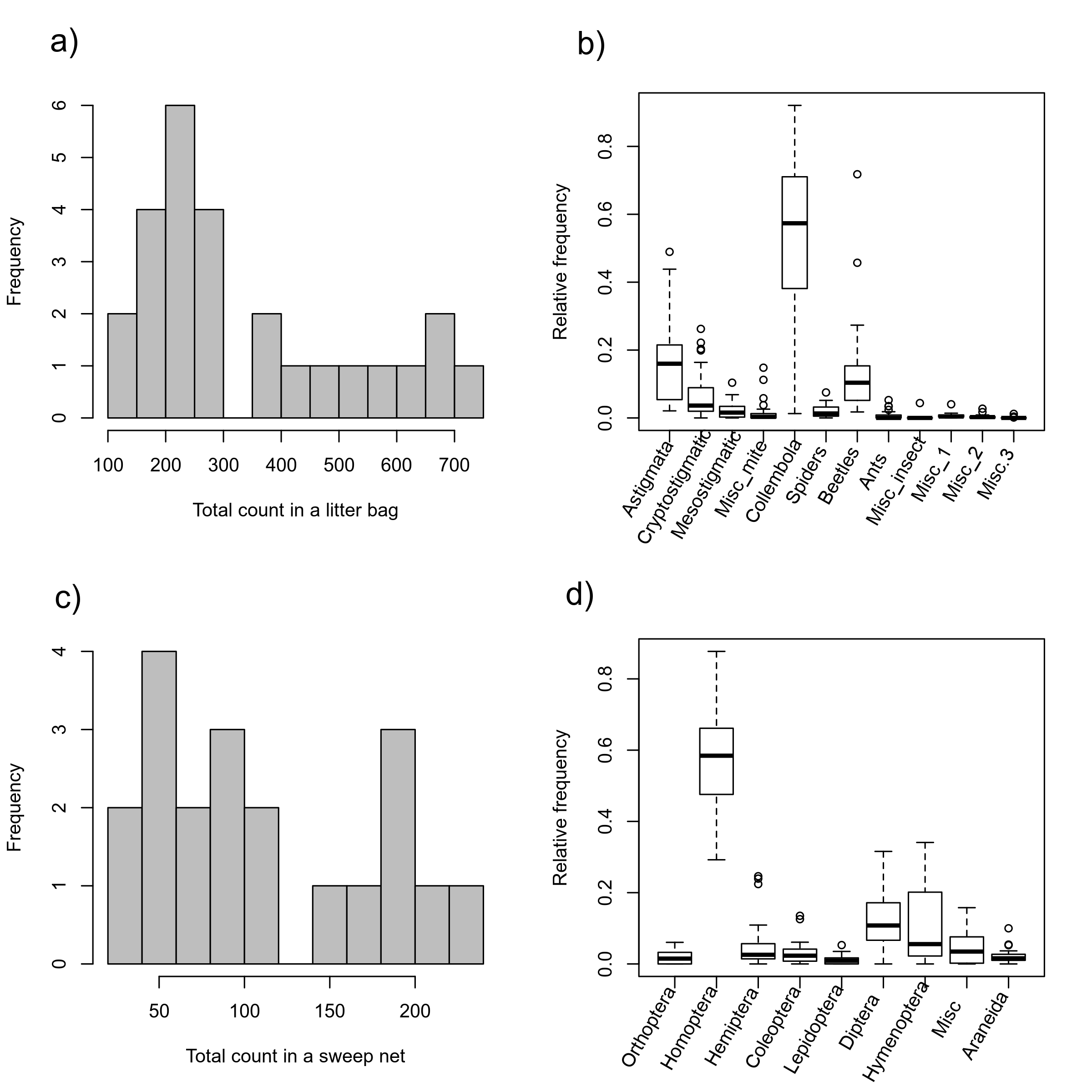


Appendix Figure 1. Summary plots of the trophic treatment in the mesocosm experiment. a) Frequency distribution of counts of litter mesofauna across representative bags sampled at the end of the experiment. b) Community composition across litter bags, identified upto order or family. Misc_mites refers to mites that are outside of the identified groups, Misc_insect are unidentified insects outside of identifiable groups and Misc_1, Misc_2 and Misc_3 are arthropods of separate, unidentified groups. c) Frequency distribution of invertebrate counts in representative sweep net drags that were used to inoculate the experimental setup. d) Community composition in sweep net drags identified upto family level where possible. Misc refers to individuals outside of identifiable groups.


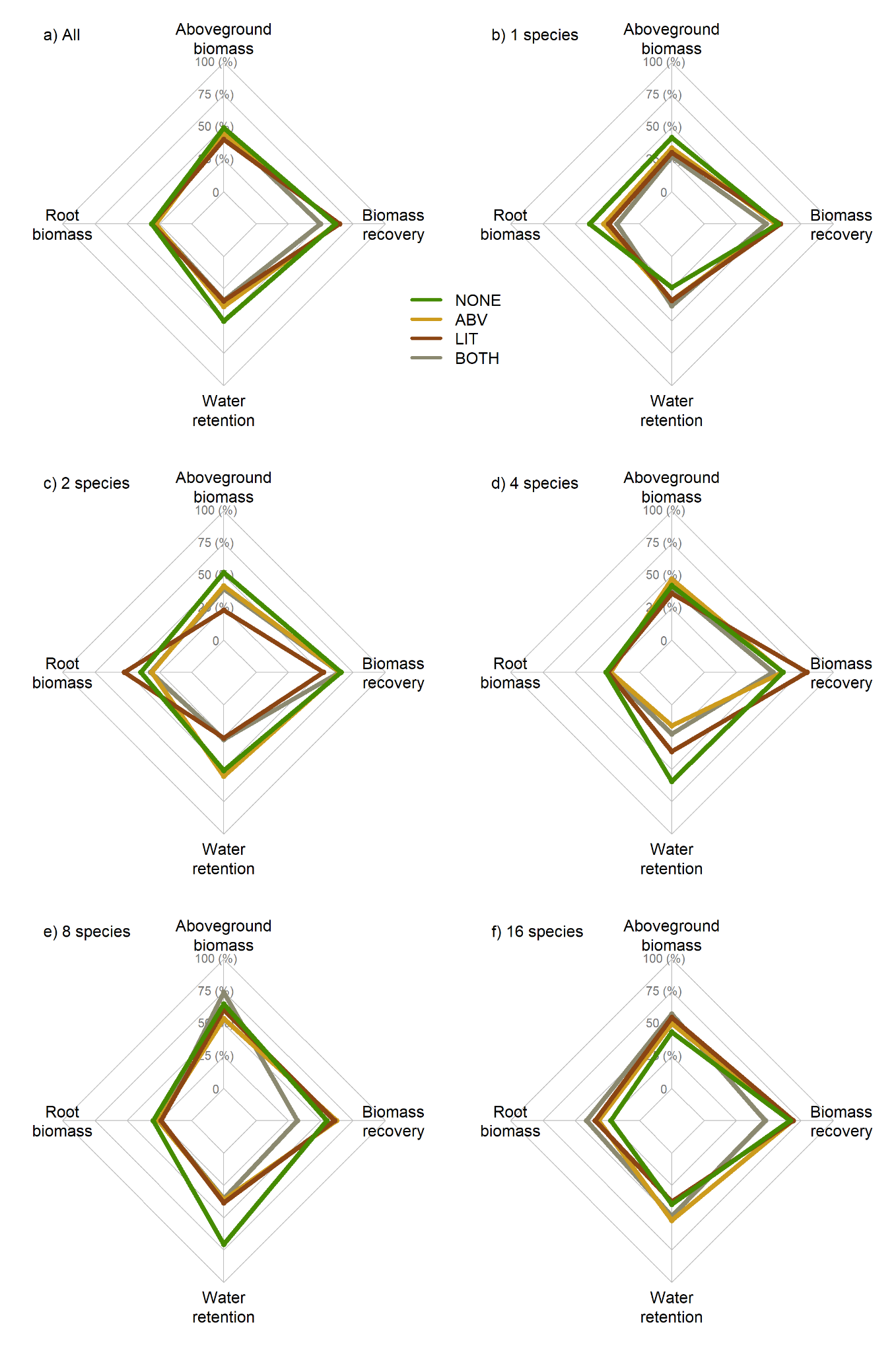


Appendix Figure 2. Spider plot showing the average percent of each function maintained by communities with different number of species and trophic complexity. a) The average percent of each function maintained by all communities in the experimental setup. The next panels represent average percent of each function maintained by communities with b) 1 plant species c) 2 plant species d) 4 plant species e) 8 plant species and f) 16 plant species. The four colors represent the four trophic treatments (plants only: NONE, plants and aboveground mesofauna: ABV, plants and litter mesofauna: LIT and plants and both aboveground and litter mesofauna: BOTH) within each plant diversity treatment. The points along each axis is calculated as the mean of each function scaled between the maximum and minimum value in the entire experiment.


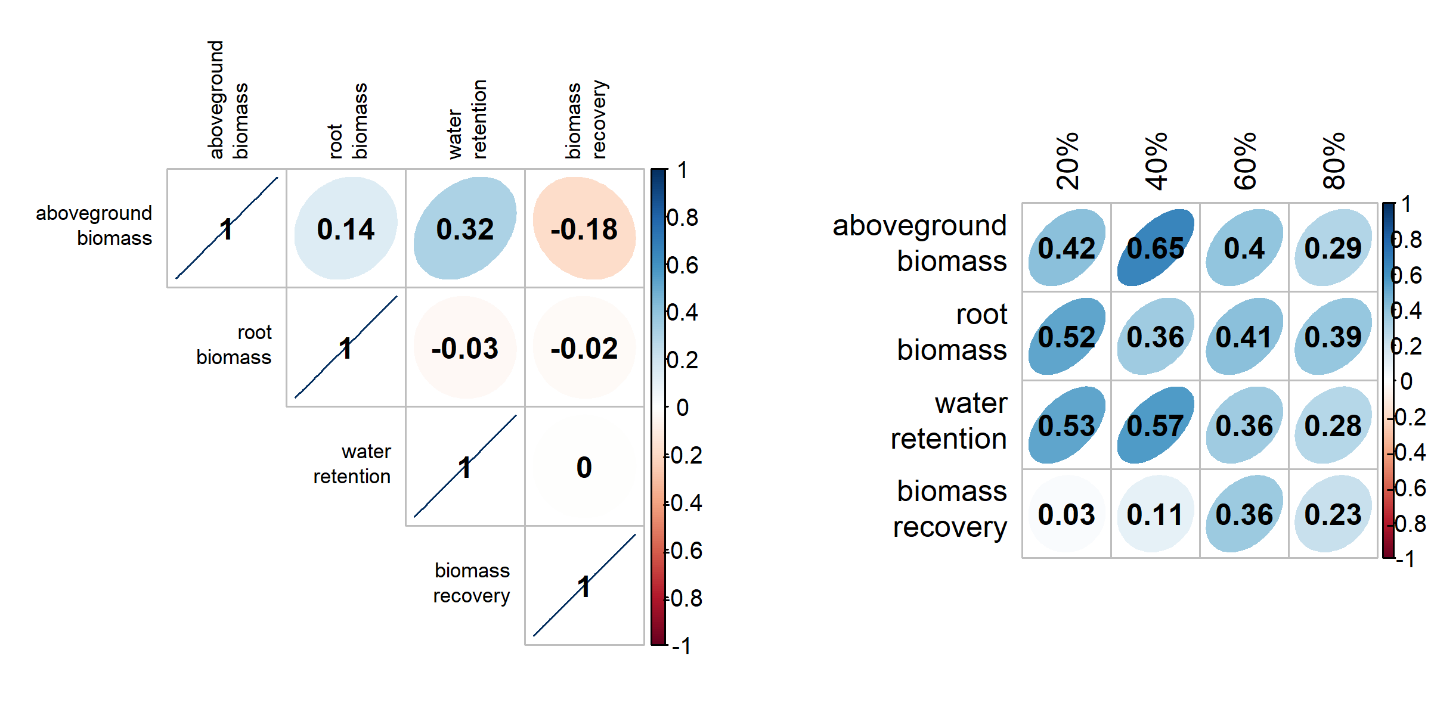


a)

b)

Appendix Figure 3. a) Correlation between the four measured functions across treatments in the experiment. Pairwise correlation coefficients are marked in each cell; and the color code is denoted in the legend. The four functions are only weakly correlated with each other at the plot level. b) The correlation between each ecosystem function measured and the multifunctionality at 20%, 40%, 60% and 80% thresholds. No single function is consistently correlated with multifunctionality of the community across thresholds and the contribution of each function to multifunctionality changes with measured threshold.
